# Real-time assessment of kidney allografts during HOPE using flavin mononucleotide (FMN) — a preclinical study

**DOI:** 10.3389/frtra.2023.1132673

**Published:** 2023-02-28

**Authors:** Richard X. Sousa Da Silva, Tom Darius, Leandro Mancina, Janina Eden, Kendra Wernlé, Ahmed S. Ghoneima, Adam D. Barlow, Pierre-Alain Clavien, Philipp Dutkowski, Philipp Kron

**Affiliations:** ^1^ Swiss HPB and Transplantation Center, Department of Surgery and Transplantation, University Hospital Zurich, Zurich, Switzerland; ^2^ Department of Surgery, Surgery and Abdominal Transplant Unit, University Clinics Saint Luc, Université catholique de Louvain, Brussels, Belgium; ^3^ Institut de Recherche Expérimentale et Clinique (IREC), Pôle de Chirurgie Expérimentale et Transplantation, Université catholique de Louvain, Brussels, Belgium; ^4^ Department of HPB and Transplant Surgery, St. James’s University Hospital, Leeds Teaching Hospitals NHS Trust, Leeds, United Kingdom

**Keywords:** kidney transplantation, hypothermic oxygenated machine perfusion, flavin mononucleotide, real-time assessment, donation after circulatory death, extended criteria donor, static cold storage, organ reconditioning

## Abstract

**Introduction:**

The gap between available donor grafts and patients on the waiting lists is constantly growing. This leads to an increased utilization of high-risk and therefore more vulnerable kidney grafts. The use of high-risk organs requires further optimization of machine preservation and assessment strategies before transplantation. Hypothermic machine perfusion (HMP) is the standard of care for kidneys originating from donation after circulatory death (DCD), whereas the evidence of HMP with additional oxygen (HOPE) is still very limited. Furthermore, an objective quality assessment of HMP-perfused kidneys is lacking. Recently, the release of mitochondria derived fragments, i.e., flavin mononucleotide (FMN) of complex I during machine liver perfusion was shown to be predictive for liver graft function before implantation. Therefore, the aim of this study was to evaluate, if FMN is useful also for assessment of kidney injury before use.

**Methods:**

A porcine perfusion model was used to investigate the feasibility of assessment of kidney grafts during hypothermic oxygenated perfusion (HOPE) with either 0, 30 or 60 minutes of warm ischemia. The model with warm ischemia times (WIT) of 30 min and 60 min, was used to mimic a clinically relevant scenario. A group with no warm ischemia time (0′ WIT) served as control group. The groups underwent minimal static cold storage (SCS) of 2 h followed by 2 h of end-ischemic HOPE with repeated real-time FMN measurements. In a further step, these values were related to the release of damage-associated molecular patterns (DAMPs) and to the functionality of the respiratory chain, represented by the capacity of ATP production.

**Results:**

We demonstrate, first, feasibility of perfusate FMN measurements in perfused kidneys, and secondly its correlation with donor warm ischemia time. Accordingly, FMN measurement showed significantly higher release in the 60-minute WIT group (*n* = 4) compared to the 30-minute WIT (*n* = 4) and the control group (*n* = 4). FMN release correlated also with DAMP signaling, such as the release of 8-OHdG and HMGB1. Finally, ATP replenishment proved to be best in control kidneys, followed by kidneys with 30 min and then by kidneys with 60 min of WIT.

**Discussion:**

This study demonstrates the feasibility of FMN measurement in kidneys during HOPE. In addition, we show a correlation between FMN quantification and pre-existing kidney graft injury. Based on this, real-time FMN measurement during HOPE may be an objective assessment tool to accept high-risk kidneys for transplantation while minimizing post-transplant dysfunction, moving away from former “gut feeling” towards objective criteria in accepting marginal kidney grafts for transplantation. Graft evaluation based on these results may close the gap between available grafts and patients on the waiting lists by increasing utilization rates without significant impact for the recipients.

## Introduction

In kidney transplantation there is a growing gap between available donor grafts and potential recipients on the waiting lists, while the demand clearly outnumbers the availability of donor organs. Efforts have been made to minimize this mismatch and further expand the deceased donor pool. Therefore, organs of extended criteria donors (ECD) and donation after circulatory death (DCD) are increasingly accepted for transplantation. Although these high-risk grafts do show similar long-term results, DCD grafts have a significantly higher occurrence of delayed graft function (DGF) compared to donation after brain death (DBD) in the short-term (1, 2). To minimize the accompanying adverse effects, dynamic preservation strategies are increasingly used for these grafts. In this respect, hypothermic kidney perfusion has shown since more than 20 years to be a superior preservation compared to static cold storage in DBD and DCD kidneys reducing delayed graft function and primary non-function rates (3, 4). Despite however this success, kidney utilization rates are still amendable and can be further increased. For example, as of March 2019, “only” 84% of kidneys offered in the United Kingdom have been transplanted (5), and criteria for organ donation remain narrow. These numbers are very similar in Switzerland (6). A potential explanation for this low utilization rate might be the ongoing lack of objective kidney viability assessment strategies for these high-risk grafts. Based on this, transplant surgeons are still reluctant in accepting high-risk kidney grafts, so called marginal grafts, which might have a negative impact on the transplant outcomes. So far, the graft evaluation and decision for organ acceptance relies on the clinical expertise of the transplant surgeon in combination with other available information (medical history of the donor, blood results, radiological imaging and preimplantation biopsy) and if necessary, the assessment of the retrieval team on site. Objective parameters for kidney acceptance or discard are still lacking.

With the widespread introduction of dynamic preservation strategies, markers in the perfusate have been assessed and evaluated during perfusion to predict organ quality. Furthermore, these markers have been correlated with post-transplant outcomes, but mainly in the field of liver transplantation. The opportunity of viability and functional assessment during organ preservation is of utmost importance to further expand the donor pool safely without compromising graft and patient outcomes. In liver transplantation, Muller et al. were able to measure Flavin mononucleotide (FMN) release in the perfusate and demonstrated a certain correlation between the extent of FMN release into perfusate and post-transplant outcomes (7).

In kidney transplantation, the assessment of objective markers during organ perfusion is still limited to preclinical studies and pilot studies in humans (5, 8). Therefore, the aim of this study was to evaluate the feasibility of FMN measurement as well as its correlation with the initial graft injury and its modulation by hypothermic oxygenated machine perfusion (HOPE).

## Methods

### Animals

Female adolescent landrace pigs weighing 47–68 kg were used for the experiments. Pigs were housed in groups of at least three and fed a regular laboratory diet for seven to ten days prior to the experiment, followed by fasting for around 6 h before the start of the experiment. Housing conditions were supervised, and all preoperative manipulations of the animals performed in-house by the veterinarians and animal caretakers in accordance with the Swiss Animal Welfare Act. The experiments were approved by the Cantonal Veterinary Office Zurich (protocol No. ZH117/2021).

### Study design and experimental groups

**Figure 1:**
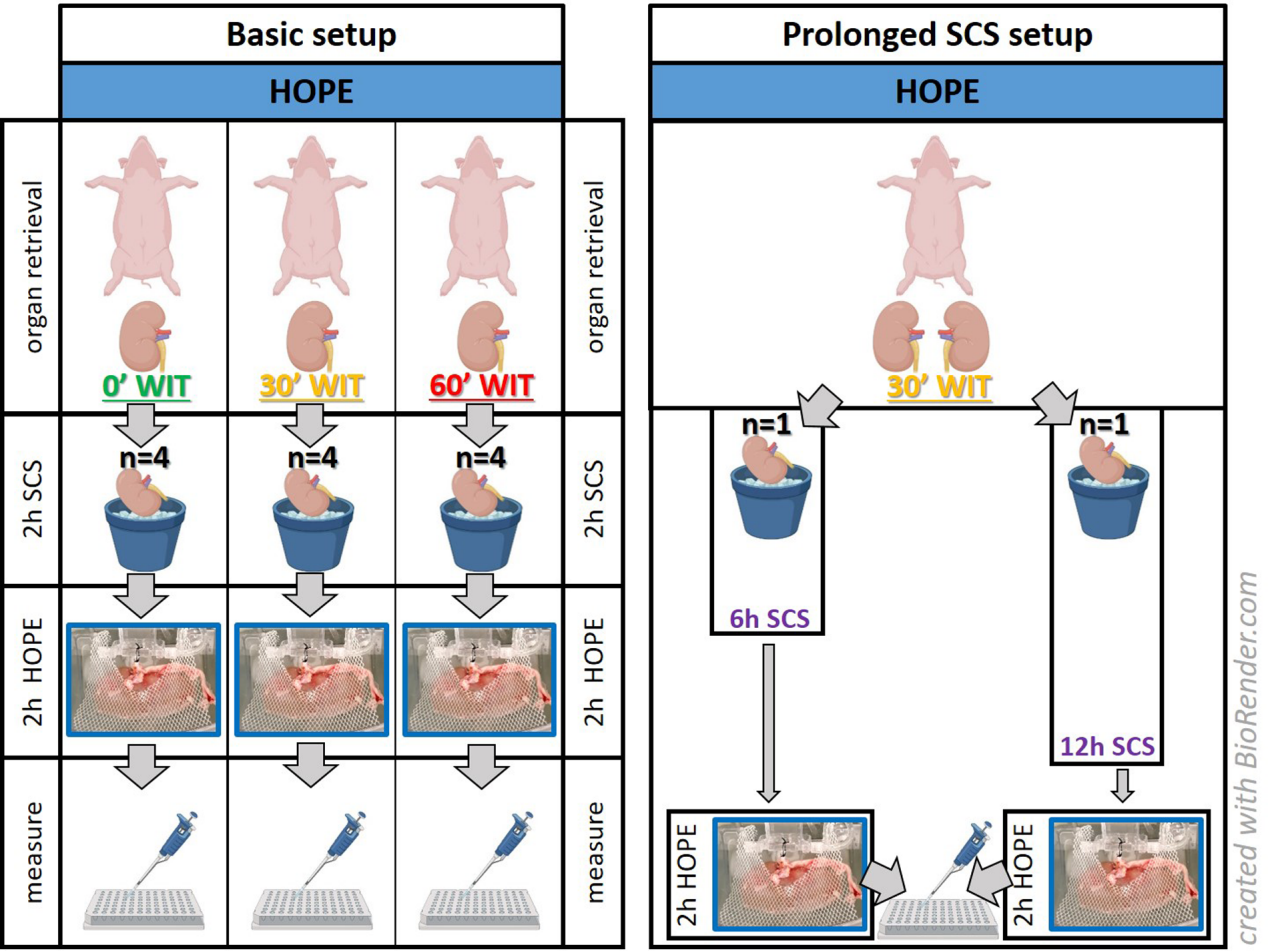
Experimental setup. The basic setup consists of kidneys (*n* = 12) studied after hypothermic oxygenated perfusion, with different initial injuries based on their warm ischemia times (WIT). A prolonged SCS setup was performed to determine whether kidneys with a 30-minute WIT showed more damage by extending static cold storage to 6 or 12 h (*n* = 2). *[HOPE = hypothermic oxygenated machine perfusion; 2 h/6 h/12 h SCS = 2 h/6 h/12 h static cold storage].*

Experiments were carried out in a 30′ and 60′ warm ischemia injury group or in a group without warm ischemia time (0′ WIT), serving as the control (Figure 1). Kidneys were retrieved after an *in situ* warm ischemia time (WIT) of either zero, 30 or 60 min depending on the study group. The aim was to correlate organ injury with FMN. Therefore, the experiments were divided into the following three main groups: a control group (minimal injury), a 30′ WIT group (intermediate injury), and a 60′ WIT group (high injury) (Figure 1). Another two additional experiments were performed in the intermediate injury group (30′ WIT) by extending the cold storage time to 6 and 12 h (Figure 1). All kidneys were subjected to two hours of hypothermic oxygenated machine perfusion (HOPE) after a previous two hours static cold storage.

### Kidney procurement and cold storage

Under general anesthesia, a midline laparotomy was performed, and kidneys were dissected free of the surrounding tissue. The hilum was then clamped, first the renal artery, then the renal vein. In the control group setting, the organ was subsequently removed within seconds by cutting the vessels and ureter followed by immediate submersion of the kidney in 2–4° cold IGL solution. In the 30′ and 60′ WIT group setting, after clamping the hilum (deprivation of the oxygenated blood supply), the kidney was left *in situ* in the pig's warm body for 30 or 60 min, depending on the targeted ischemic injury. After retrieval, all kidneys were rinsed with 500 ml of chilled Ringer before being flushed with 1 liter of IGL for two hours of storage. Determination of which kidney was used for which study group was randomly assigned.

### Hypothermic oxygenated perfusion (HOPE)

The retrieved kidney was prepared for perfusion by connecting the renal artery with a straight 3-mm cannula before connecting to the LifePort® Kidney Transporter perfusion circuit (Organ Recovery Systems, Itasca, IL, United States) (Supplementary Figure S1). The circuit's 1-liter Belzer MPS® perfusion solution was previously saturated with oxygen (oxygen air bubbling) for 20 min, achieving a O_2_ partial pressure of 70–90 kPa. During two hours of hypothermic oxygen perfusion, perfusate samples were collected at designated time points, i.e., after 5, 10, 15, 30, 60, and 120 min.

### Endpoints

The primary endpoint was measurement of flavin mononucleotide (FMN) concentration over time during machine perfusion, indicating the degree of mitochondrial damage (respiratory chain). Additional endpoints included analysis of energy levels *via* ATP and further assessment of the initial injury during perfusion *via* damage-associated molecular patterns (DAMPs).

### Quantification of FMN, qPCR, ELISAs and statistical analysis

Flavin mononucleotide concentration was measured by fluorescence spectroscopy with Cytation Reader 3 (BioTek Instruments, Winooski, VT, United States) based on emission intensity at 528 nm after excitation with light at 485 nm. Therefore, 50 μl of each sample was added to 150 μl of 0.9% NaCl in a 96-well plate. To verify that FMN and not another substance was truly measured in the perfusate, a standard solution containing pure FMN at a concentration of 0.3 µg/ml was added to the samples to detect a uniform increase in offset across all samples (Supplementary Figure S2), corresponding to the value of the standard solution. A correlation curve between FMN concentration and fluorescence measurement (in arbitrary units, A.U.) proved a linear correlation (R^2 ^= 0.9987) (Supplementary Figure S3). In the different injury groups a correlation of the mitochondrial and perfusate FMN content was done *via* liquid chromatography-mass spectrometry (LC-MS) (Figure 4). Mitochondrial isolation was performed according to the group of Galkin et al. (9).

DAMPs in the tissue were measured using 7,500 Fast Real-Time PCR System (Applied Biosystems, Waltham, MA, United States) and induction fold normalized to 18S expression. Enzyme-linked immunosorbent assays were performed to measure perfusate 8-OHdG (MBS269139, MyBioSource, San Diego, CA, United States) and HMGB1 (ST51011, IBL International, Hamburg, Germany) levels. Data analysis and charting were performed using GraphPad Prism 9 (GraphPad Software, United States). Data are presented as median and interquartile range, and group comparison was performed with a 2-way ANOVA test.

## Results

### Effect of injury severity on FMN release

**Figure 2:**
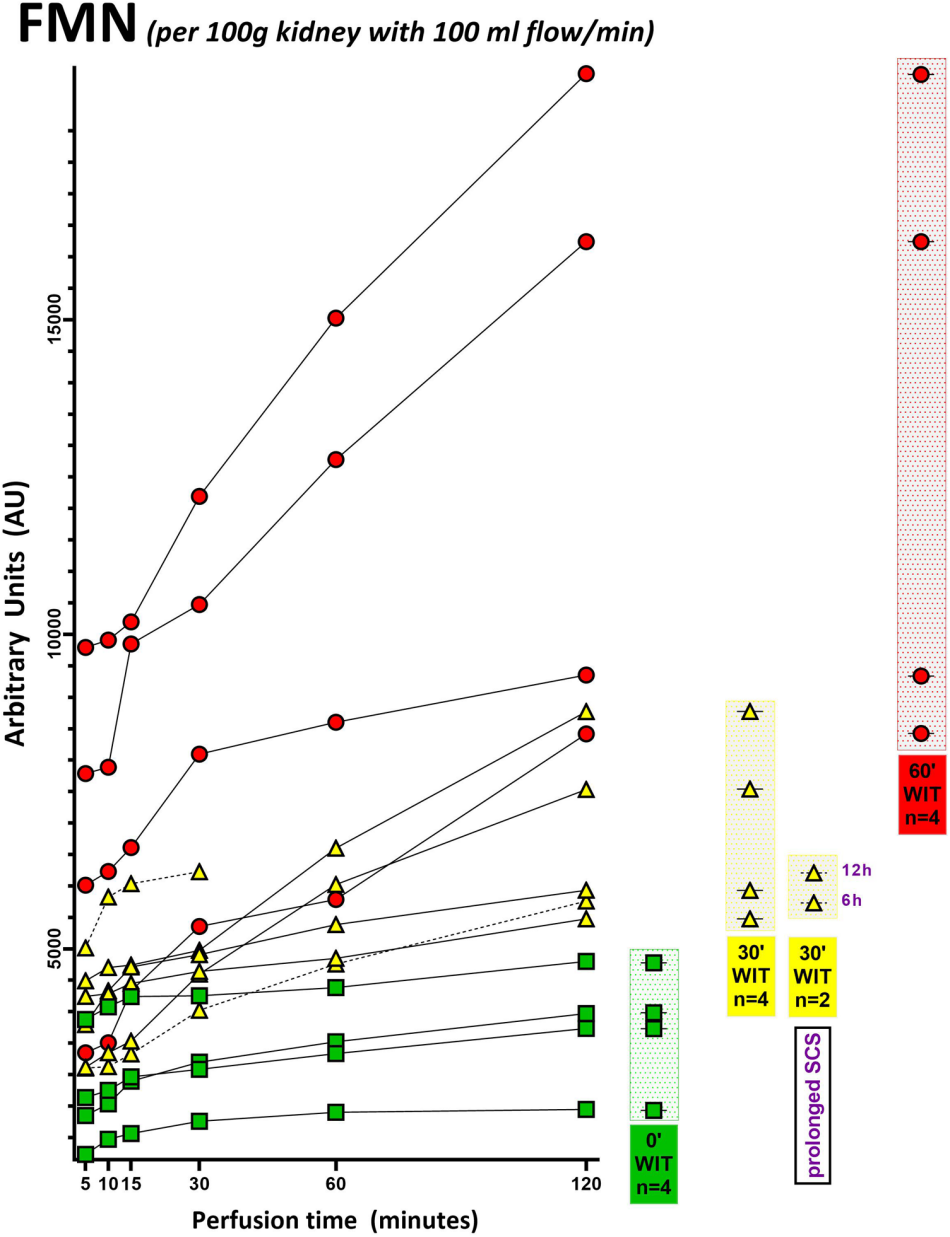
FMN measurement of each porcine kidney during 120 min of hypothermic oxygenated perfusion (HOPE). All FMN values (arbitrary units) are normalized to kidney weight and perfusion flow. Green icons represent no warm ischemia time (WIT) (0′ WIT = control) kidneys, whereas yellow icons represent kidneys with 30 min of warm ischemia time (30′ WIT) and the red ones those with 60 min of warm ischemia time (60′ WIT). *[6 h = 6 h static cold storage; 12 h = 12 h static cold storage].*

**Figure 3:**
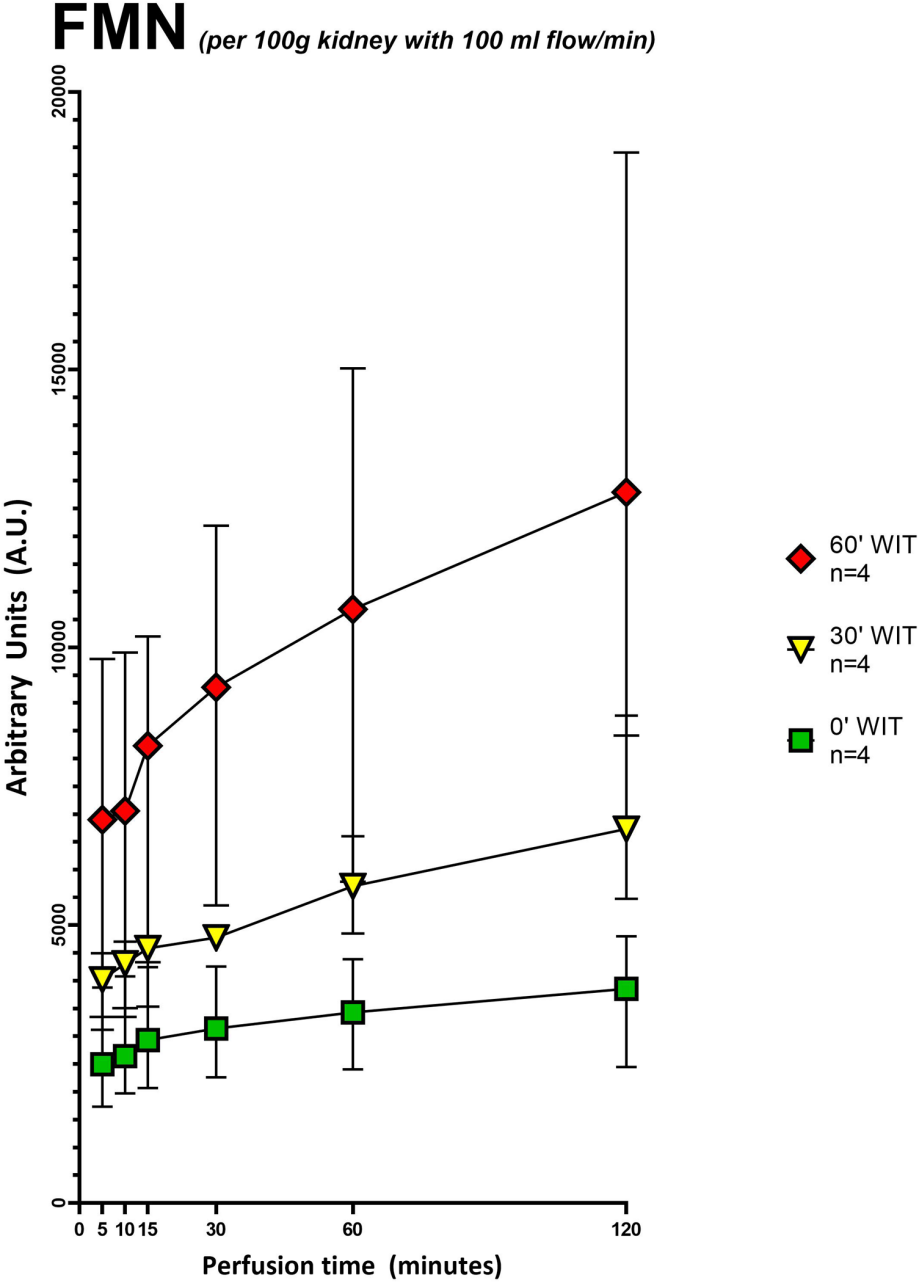
Grouped FMN values (arbitrary units) during 120 min of hypothermic oxygenated perfusion (HOPE). Each group consists of pig kidneys belonging to either the no warm ischemia time (WIT) (0′ WIT = control), the 30 min warm ischemia time (30′ WIT) or 60 min warm ischemia time (60′ WIT) group. Those with extended storage time (6 and 12 h) were not included. *[Icons indicate median and whiskers the interquartile range].*

Fourteen porcine kidneys were placed on HOPE, with subsequent analysis of perfusate and fresh frozen tissue samples. The first aim was to demonstrate successful detection of mitochondrial FMN release during HOPE. In all groups, independent of the initial injury of the organ, FMN could be detected. Furthermore, measured FMN levels increased over time during perfusion (Figures 2, 3). This can be explained at the molecular level, as oxygenation of ischemic tissue results in generation of reactive oxygen species (ROS) (Figure 7). One of the main drivers of this effect is the accumulation of the tricarboxylic acid (TCA) metabolite succinate. In addition, no significant increase in FMN release was observed when the SCS was extended to 6 or 12 h, consistent with the safe clinical use of static cold-stored kidneys for up to 12 h prior to transplantation.

**Figure 4:**
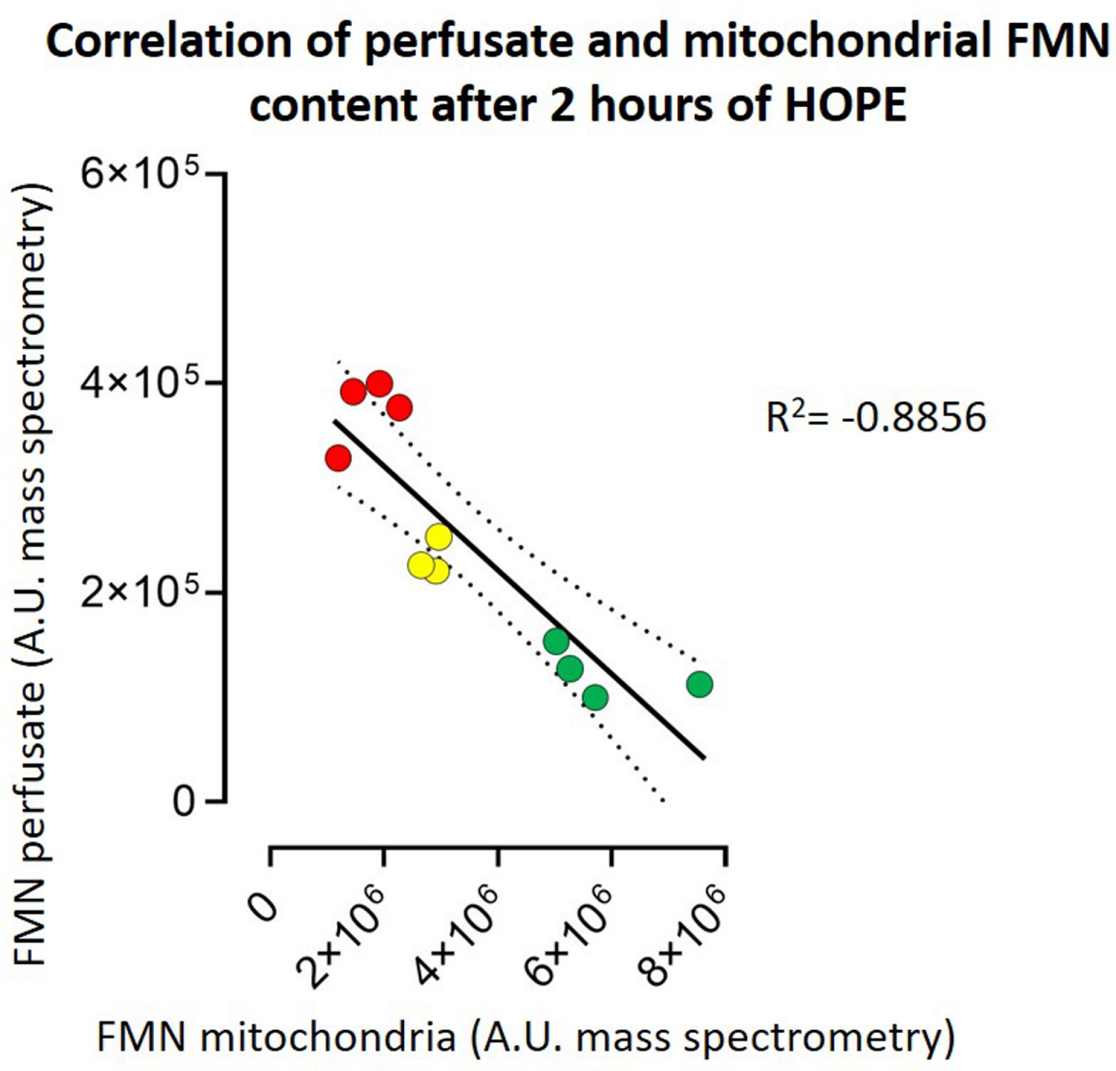
Correlation of perfusate and mitochondrial FMN content after 2 h of HOPE. The higher the FMN content in the perfusate, the lower the FMN content in the mitochondrial membrane—both measured in arbitrary units (A.E.) by mass spectrometry. This results in a correlation R^2^ of −0.8856. *[red symbols = kidneys with 60 min warm ischemia time (60, WIT) (n = 4); yellow symbols = kidneys with 30 min warm ischemia time (30, WIT) (n = 3); and green symbols = kidneys without warm ischemia time (0, WIT) (n = 4)].*

Our second aim was to investigate the total amount of FMN detected during perfusion and its correlation with the different underlying injuries of the grafts. FMN was normalized to kidney weight and flow (Supplementary Table S1). The flow, as usually in kidney perfusion, was pressure regulated with a standard pressure of 30 mmHg. When normalized to kidney weight (125–192 g) and perfusion flow (29.6-79.5 ml/min) FMN showed a significant discrepancy between the different injury groups (Figure 3). Namely kidneys with 60-minutes of warm ischemia time (WIT) had the highest values for FMN, followed by lower values in organs with 30-minutes of WIT and in the control organs (*p* < 0.05) (Figure 3). Moreover, LC-MS showed a linear negative correlation (R^2^ = −0.8856) between FMN release into the perfusate and the amount of FMN remaining in mitochondria at the end of HOPE (Figure 4).

This observation proves that the released FMN originates from mitochondria. Therefore, kidneys with higher injury levels (60′ WIT > 30′ WIT > control) and higher amounts of FMN in the perfusate have lower amounts of FMN in the mitochondria (Figure 4).

**Figure 5:**
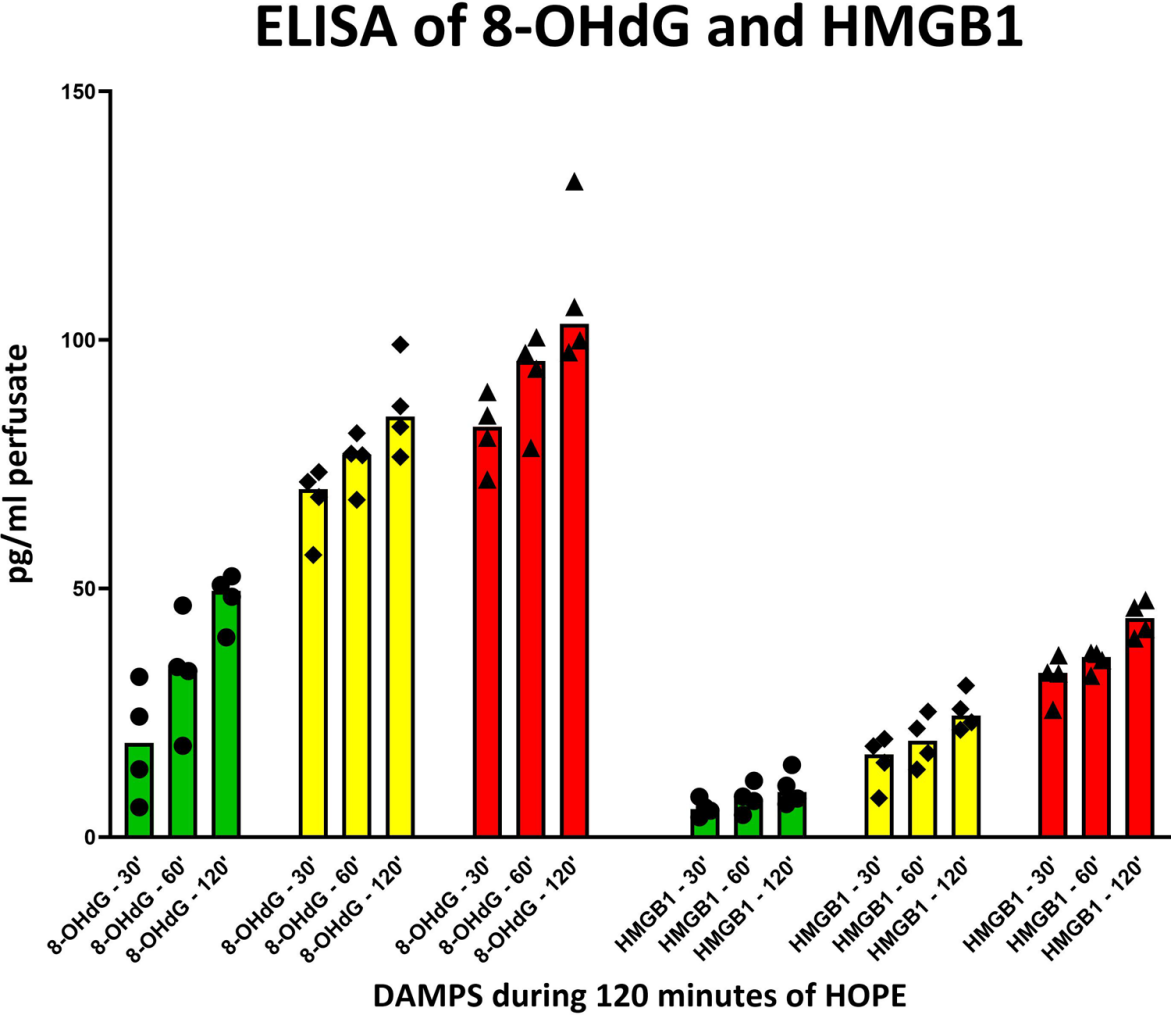
8-OHdG and HMGB1 release in perfusate during 120 min of hypothermic oxygenated perfusion (HOPE). Kidneys with higher initial injury depict higher release of these two damage-associated molecular patterns (DAMPs*). [Each group consisting of 4 kidneys; green = 0, WIT, yellow = 30, WIT; red = 60, WIT].*

Similar to FMN, DAMPs, 8-OHdG and HMGB1 allowed a clear discrimination between the different underlying injury groups, with the highest concentration levels found in the 60′ WIT group, followed by 30′ WIT group and the lowest levels in the control group without any warm ischemia time (Figure 5).

### Protective effect of HOPE

**Figure 6:**
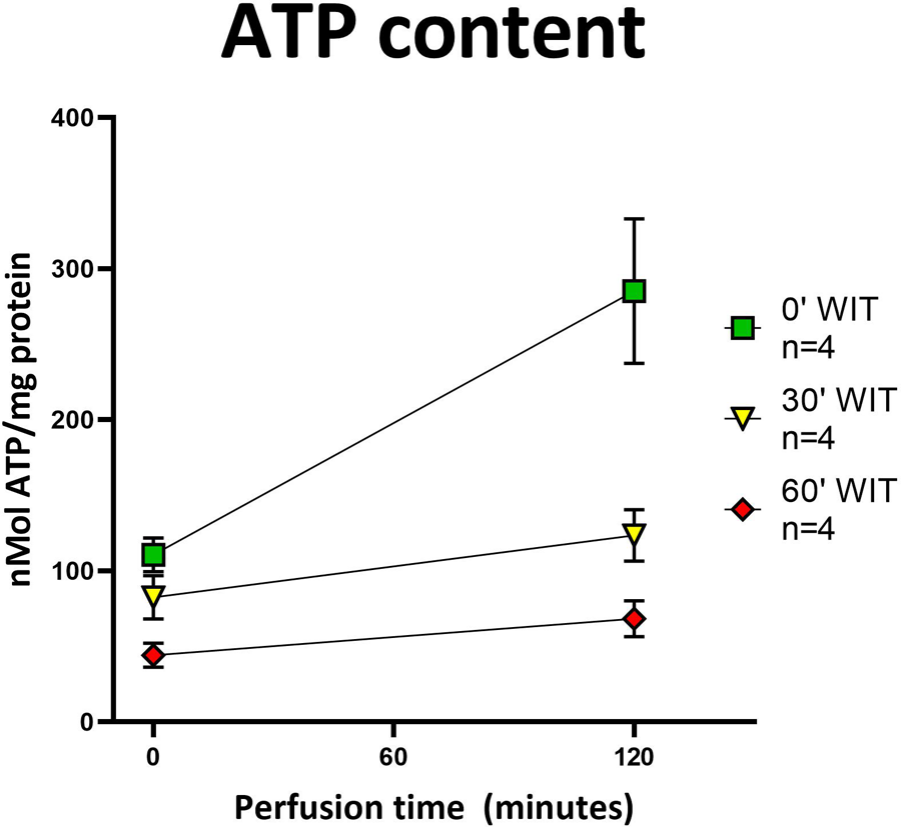
ATP content in tissue before and after HOPE. All three groups showed an increase in ATP content after HOPE compared to before. Organs without warm ischemia time (0′ WIT) regained the most ATP, starting with the highest values. Organs with 30′ WIT and 60′ WIT started with lower values and did not recover ATP to a similar extent. *[Icons indicate median and whiskers the interquartile range].*

The protective effect of HOPE was demonstrated in this perfusion setting of porcine kidneys, evidenced with the successful replenishment of ATP stores during perfusion (Figure 6). In addition, the more extensive the initial damage was (equal to the warm ischemia time), the lower the ATP levels at baseline were found to be (60′ WIT group <30′ WIT group < control group). Moreover, the kidneys in the control group replenish most ATP by the end of the HOPE period, whereas kidneys in the 30′ WIT group and even more severely damaged kidneys with 60 min of WIT produce the least ATP (Figure 6).

Reperfusion-induced exacerbation and perpetuation of the initial injury can be identified by the release of DAMPs, latter downstream targets in the ischemia-reperfusion cascade. Notably, analysis of DAMPs such as 8-OHdG and HMGB1 in the HOPE perfusate, like FMN, allowed clear discrimination between the different injury groups, with the highest levels found in the group with 60′ minutes of warm ischemia (Figure 5).

## Discussion

This is the first experimental study demonstrating that the extent of FMN release in the perfusate during hypothermic oxygenated kidney machine perfusion is in correlation with the initial underlying graft injury. The results indicate differences between kidneys without any additional warm ischemia time and kidneys with substantial warm ischemia time of 30′ and 60′ minutes. These differences are displayed in terms of FMN measurements in the perfusate. The active oxygenation during HOPE results in a forward electron transfer keeping FMN levels low with replenishment of ATP and higher energy levels at the end of preservation. Furthermore, results of latter downstream targets are in line with the FMN values measured. Based on these results and given the evidence that FMN correlates with outcomes after liver transplantation (7), this may provide a novel and objective assessment tool for kidneys during HOPE in the future (8). Next, the possibility of real-time measuring FMN during HOPE shows that viability assessment during cold perfusion is feasible. For a long time, it was postulated that viability and graft function assessment is only possible during normothermic machine perfusion (10, 11). By mimicking a “near physiological state”, it was more comprehensible that assessment of organ function is feasible and more logical during normothermic organ perfusion (11–13). The lack of graft assessment was claimed to be one of the major shortcomings during hypothermic machine perfusion approaches. Muller et al. were the first to show that viability assessment during hypothermic oxygenated liver machine perfusion is possible. In their study, they could show that measurement of FMN during HOPE of liver allografts is not only feasible but even can be used as a predictive surrogate marker for post-transplant outcomes. “Classic markers” such as lactate or transaminases and donor and recipient risk scores were ineffective in predicting outcomes after transplantation, whereas FMN was the only biomarker that could show a robust correlation (7). Their study demonstrated that FMN measurements correlated with hospital stay, cumulative complications and 90-day graft loss of the recipients (7). Furthermore, they could show that FMN measurement *via* fluorescence spectroscopy is a real-time graft assessment that is practical, inexpensive, and most importantly, objective and reproducible. These findings were confirmed by other groups in the clinical setting of liver transplantation (14, 15). In kidney graft evaluation, this confirmation has eagerly been awaited but the validation of this approach is relatively new and limited to preclinical models in porcine kidneys during hypothermic perfusion (5, 8). In the “normothermic setting”, there are pilot studies available assessing FMN concentrations during normothermic regional perfusion (NRP) and normothermic machine perfusion (NMP) of kidney grafts (5). The Cambridge group could show that the levels of FMN were significantly higher in most grafts (7/11; 63.6%) that developed DGF or PNF (5). Their results are in line with existing evidence showing that FMN can be correlated with post-transplant graft outcomes. Since this was a pilot study, their cohort was too small to further evaluate any threshold for acceptance or discard of kidney grafts. Darius et al. could show in their study that FMN measurement is also possible during hypothermic oxygenated perfusion of porcine kidney grafts and that the extent of FMN release correlates well with initial graft function after transplantation (8).

In our study, FMN measurement was for the first time correlated with the underlying organ injury. To further strengthen this theory latter downstream targets in the ischemia-reperfusion cascade were measured on protein level. They also showed a gradual increase depending on the underlying graft injury (control <WIT 30′ <WIT 60′) confirming the distinction and correlation of FMN with the underlying graft injury. This correlation of FMN release with the underlying graft injury was also shown by other groups, however in livers and not in kidneys (7, 14). The source of the flavin in the perfusate is worth discussing. There are 76 flavoenzymes in the mammalian proteome, only 12 bearing FMN (16). Potentially all the enzymes with non-covalently bound cofactor can be a source of the flavin, which might dissociate in the reductive condition of ischemia. Dissociation of the reduced flavin from flavoenzymes was observed *in vitro* other enzymes (17–19) and recently detected *in vitro* and *in vivo* for mitochondrial complex I (9, 20, 21). Complex I of the inner mitochondria membrane is solely responsible for NADH oxidation in the matrix during the entry step of electron transfer in the respiratory chain and contributes significantly to formation of proton gradient for ATP synthesis by transporting protons to the intermembrane space (22–24). FMN is tightly and non-covalently bound to complex I hydrophilic domain. During physiological cell redox status, complex I-bound FMN is in a stable junction with the apoenzyme. However, after reduction during ischemia followed by reperfusion, a certain flavin-release is triggered (9, 25). Due to this ischemia-induced reductive dissociation of FMN and high abundance of mitochondrial complex I in kidneys (26), it is very likely that flavin detected in perfusate is liberated from that enzyme, rendering it inactive. The importance of mitochondrial function and the strong association with energy production is very well known, but the impact of FMN as an objective marker for graft viability assessment and the predictive value for post-transplant outcomes is relatively new (27). Not only could FMN be correlated with post-transplant outcomes, but the authors were also able to define a clinically relevant threshold for liver transplant acceptance and rejection. In renal transplantation, there are only pilot human and preclinical studies that provide unreliable markers for outcome correlation, such as lactate. Therefore, thresholds for accepting or declining kidney grafts have not yet been established.

High volume clinical trials are however needed to establish such cut-off values, and the focus should be on reliable and promising markers as FMN. The two recently published RCTs in kidney transplantation under the patronage of the Consortium for Organ preservation in Europe (COPE) were investigating the effect of HOPE. One trial compared upfront addition of oxygen during hypothermic perfusion to regular HMP in DCD kidneys, while the other trial investigated the effects of end-ischemic HOPE after static cold storage (SCS) vs. SCS alone in ECD grafts (28, 29). Husen et al. could not show a benefit for end-ischemic HOPE compared to SCS alone in regards to graft survival or graft function (28). However, the end-ischemic perfusion period was limited to 2 h, which might be too short and could potentially explain the lack of benefits in the HOPE group (30). Jochmans et al. could show a significant reduction of post-transplant complications in kidney grafts being treated with upfront HOPE. Furthermore, they could observe a 44% relative risk reduction in acute rejection. This interaction of HOPE and the resulting effect of reduced activation of the immune response in recipients has been observed before (31).

**Figure 7:**
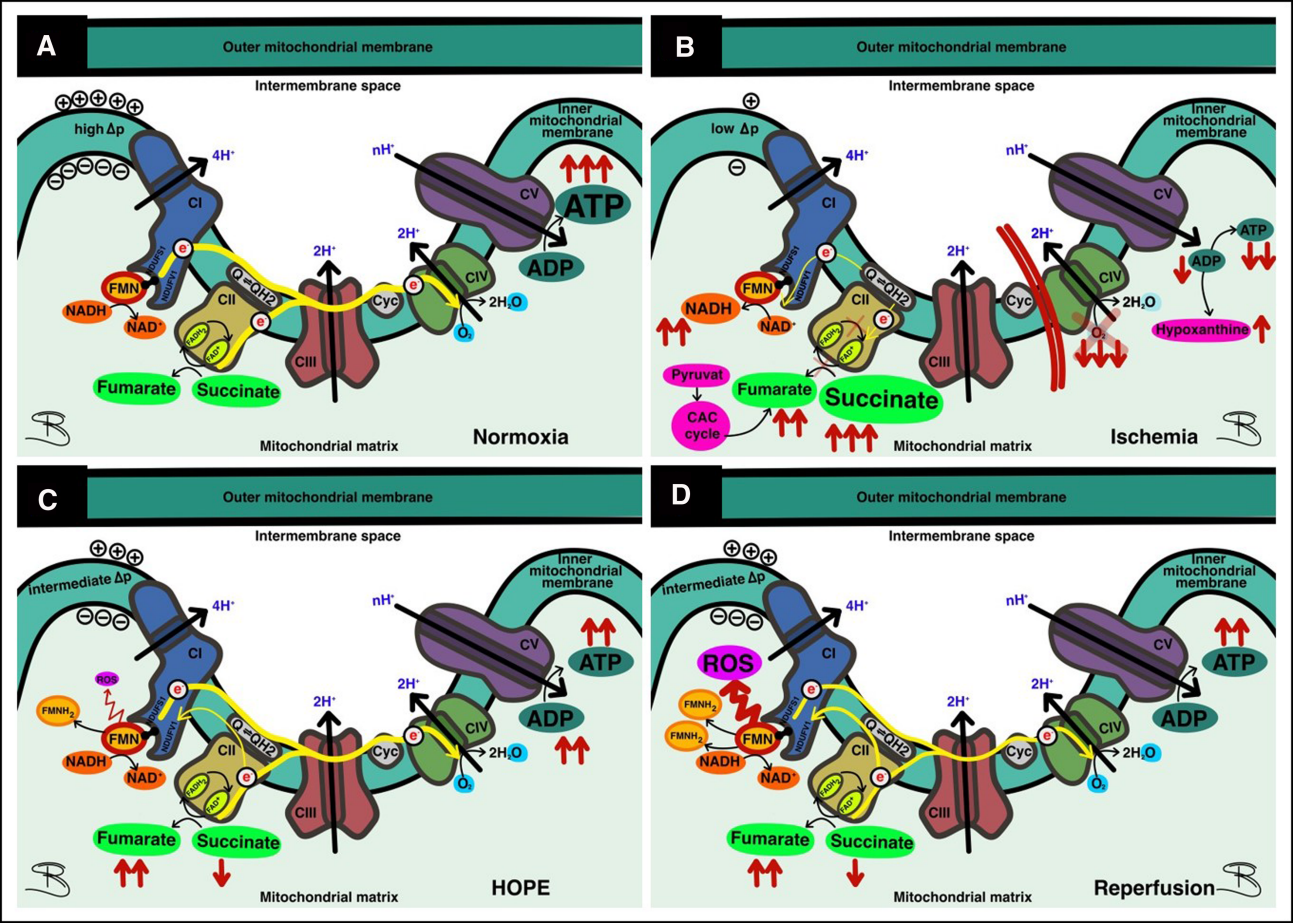
Function and dysfunction of the respiratory chain. (**A**) At normoxia forward electron transport (ET) provides ATP production at complex V; (**B**) In ischemia, ET is impaired due to oxygen deprivation, leading to accumulation of succinate; (**C**) Under hypothermic oxygenated perfusion (HOPE), most ET occurs in the forward direction, allowing ATP replenishment—some reverse electron transport (RET) results in damage to complex I with FMN release; (**D**) At reperfusion, where high metabolic activity prevails, massive RET results in complex I injury and concomitant FMN release.

Since the kidney, like the liver and heart, is an organ with high mitochondrial content, the effects of HOPE should also be seen here in a manner similar to liver perfusion (32). In this study, hypothermic oxygenated perfusion prevented a certain reperfusion injury with less FMN release and decreased activation of latter downstream targets (DAMPs). Furthermore, the ATP levels increased after HOPE in all injury groups compared with baseline values. These results clearly show a desirable reduced metabolism under the protection of hypothermia and confirm that HOPE is able to induce a mitochondrial switch, reloading the cellular ATP battery by fueling proton pumps and ATP synthase (Figure 7). Kidney cells can therefore be recharged during perfusion without any significant damage.

In all studies in which FMN was evaluated, real-time measurements were performed by fluorescence spectrometry (7, 25, 33). This proved to be a simple and economical method to measure FMN. Moreover, the results are available on site within minutes allowing a “real-time” assessment of grafts. In the study by Wang et al., FMN content in the blood-based perfusate was also measured by fluorescence spectrometry (5). It would have been important to confirm these measurements *via* mass spectrometry to be sure that the detected marker in the sample is FMN. Compared to NMP, this is different in cold, as acellular perfusate is used for hypothermic machine perfusion. We could show a nearly perfect linearity between FMN concentration and increase of arbitrary units in the fluorescence spectrometry (Supplementary Figure S2). To be sure to measure FMN, a fixed known amount of FMN was added to the perfusion fluid samples. In a second step the FMN content in these samples was measured again, showing that the difference between these two measurements was the amount of FMN added (Supplementary Figure S3). For further confirmation, FMN liquid chromatography-mass spectrometry was performed externally. This examination could prove that FMN was measured. Furthermore, it could show a redistribution of mitochondrial FMN depending on the underlying injury of the kidney graft. Figure 4 shows a linear negative correlation of FMN release into perfusate and the amount of remaining FMN in the mitochondria in the different injury groups.

This study is limited by the absence of a transplantation- or *ex situ* reperfusion model. However, the correlation of HOPE with post-transplant outcomes has been confirmed in kidney transplantation as well as in other organs before (7, 8). Since the introduction of FMN and DAMPs is relatively new in the assessment of graft injury before transplantation, it would be highly interesting to correlate the coherence of these new markers with more classical markers for outcome after kidney transplantation, such as the Remuzzi Score, in future studies.

We believe that the findings of this study are of importance and have the potential to change the clinical routine of organ allocation. In the clinical setting, the introduction of FMN real-time measurement in combination with HOPE might allow us to accept and decline kidney grafts based on objective parameters. Furthermore, a tailored perfusion approach can be offered, considering the individual needs of every graft based on these measurements, such as the optimal duration of perfusion required. Therefore, we envision a further expansion of the donor pool with high-risk grafts and increase utilization rates safely.

## Conclusion

In summary, this study confirms the feasibility of FMN measurement during HOPE, and the correlation of this biomarker with initial graft injury. At this point, further research is crucial to validate and explore the role of biomarkers in organ assessment, and more importantly, to define thresholds for acceptance and discard. With further validation, FMN could serve as a novel biomarker also for kidneys before implantation moving away from the earlier “gut feeling” to objective criteria.

## Data Availability

The raw data supporting the conclusions of this article will be made available by the authors, without undue reservation.
