# Supplementary material for: Real-time assessment of kidney allografts during HOPE using flavin mononucleotide (FMN) — a preclinical study
Source: Front Transplant. 2023 Feb 28;2:1132673. doi: 10.3389/frtra.2023.1132673 (PMC11235286; doi:10.3389/frtra.2023.1132673)
Supplement: Supplementary file 1 [file Datasheet1.pdf]

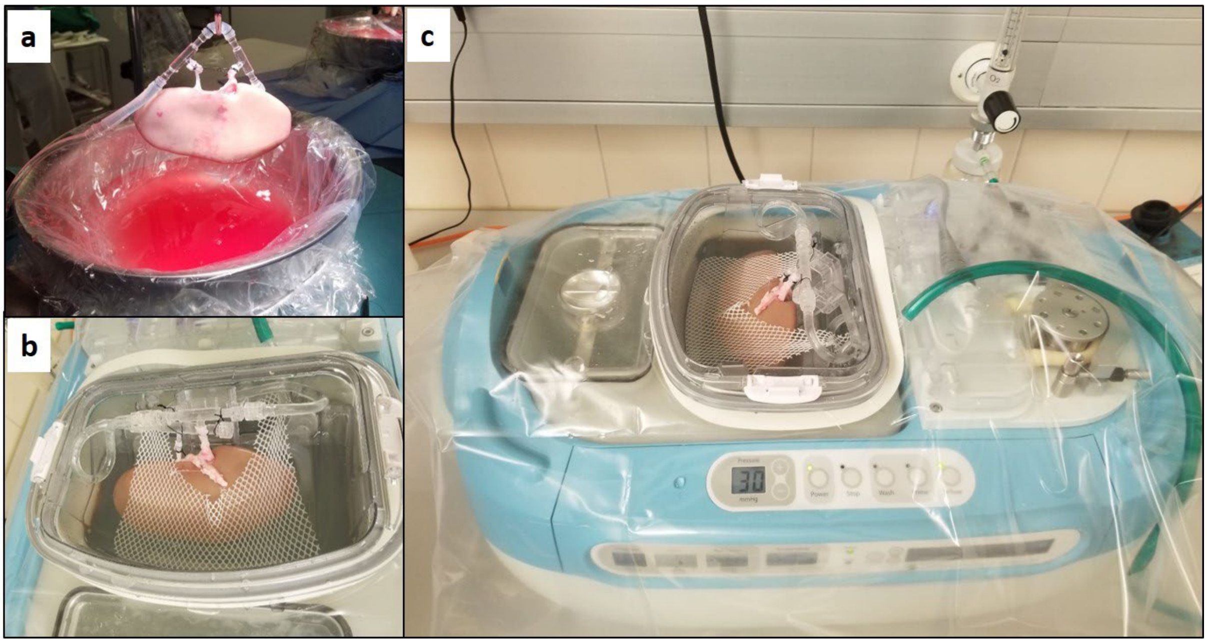

SUPPLEMENTARY FIGURE 1. Hypothermic oxygenated perfusion (HOPE) of porcine kidneys. a) kidney being rinsed with 500 ml of chilled Ringer after cannulation; b) connection of kidney to the LifePort®Kidney Transporter perfusion circuit (Organ Recovery Systems, Itasca, IL, USA); c) kidney during 120 minutes of perfusion in the LifePort®Kidney circuit loaded with 1 liter Belzer MPS® perfusion solution.

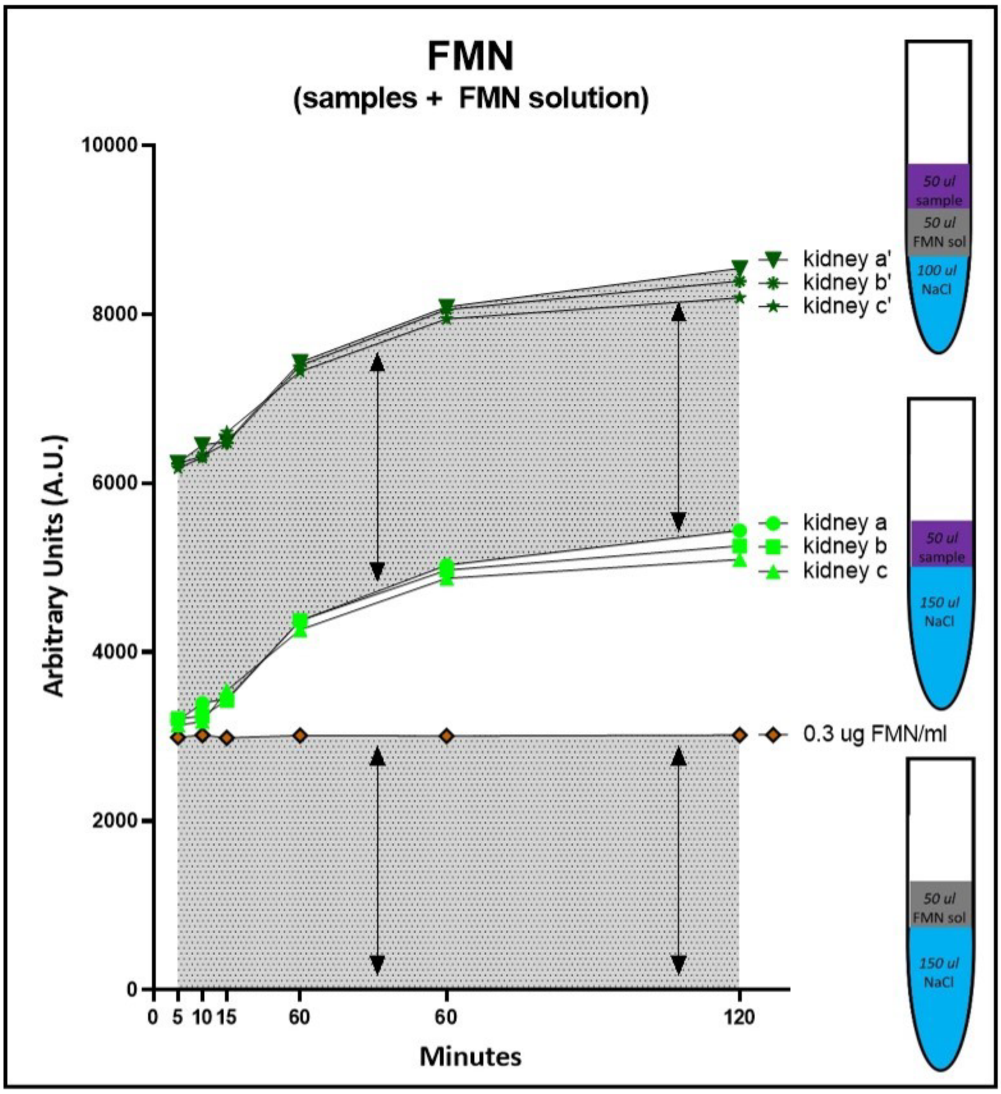

SUPPLEMENTARY FIGURE 2. Twofold FMN measurement (A.U.) of three kidneys with and without added FMN solution. FMN solution (0.3 µg/ml) had a mean A.U. of 3000. When added to the perfusates of kidneys a-c, the readings of the new samples (kidney a'-c') showed an increase in A.U. that exactly matched the FMN solution.

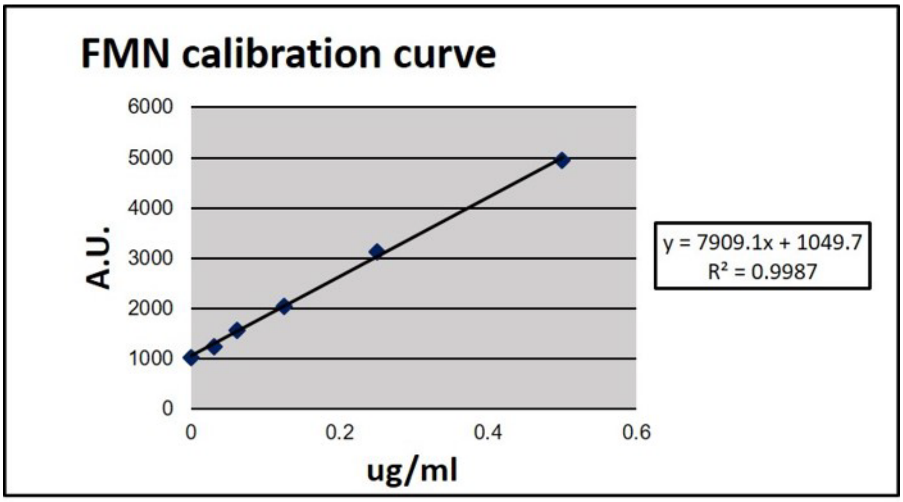

SUPPLEMENTARY FIGURE 3. FMN calibration curve. A flavin mononucleotide solution of 0.5 µg/ml was prepared and diluted to 0.25, 0.125, 0.0625, and 0.03125 µg/ml to be measured by fluorescence spectroscopy, which showed a robust linear correlation between concentration and arbitrary units detected (R2=0.9987).

Kidney graft and perfusion characteristics						
**Injury group**	**Kidney**	**Weight of kidney graft (g)**	**Mean perfusion flow (ml/min)**	**Duration of static cold storage (hours)**	**Set perfusion pressure (mmHg)**	**Perfusion fluid (l)**
0' WIT	1	152	76	2	30	1 MPS Belzer®
	2	153	79.5			
	3	165	68.3			
	4	167	70.6			
30' WIT	5	157	53			
	6	155	52.6			
	7	166	54			
	8	162	50.4			
	9	184	49.8	6		
	10	192	48	12		
60' WIT	11	125	30.5	2		
	12	149	29.6			
	13	167	39.4			
	14	182	40.8			

SUPPLEMENTARY TABLE 1. Characteristics of the kidney grafts and the perfusion. Weight of the kidney graft and mean perfusion flow was used to assess weight- and flow-normalized FMN curves (Figure 2). Perfusion pressure was set at 30 mmHg. Each HOPE perfusion used 1 liter of MPS Belzer®.
